# Analysis of Expressed Sequence Tags from Chinese Bayberry Fruit (*Myrica rubra* Sieb. and Zucc.) at Different Ripening Stages and Their Association with Fruit Quality Development

**DOI:** 10.3390/ijms14023110

**Published:** 2013-02-01

**Authors:** Changqing Zhu, Chao Feng, Xian Li, Changjie Xu, Chongde Sun, Kunsong Chen

**Affiliations:** Laboratory of Fruit Quality Biology/The State Agriculture Ministry Laboratory of Horticultural Plant Growth, Development and Quality Improvement, Zhejiang University, Zijingang Campus, Hangzhou 310058, China; E-Mails: zcq1236@zju.edu.cn (C.Z.); 11016044@zju.edu.cn (C.F.); xianli@zju.edu.cn (X.L.); chjxu@zju.edu.cn (C.X.); akun@zju.edu.cn (K.C.)

**Keywords:** Chinese bayberry (*Myrica rubra* Sieb and Zucc.), fruit ripening, expressed sequence tags (EST), fruit quality, development, senescence

## Abstract

A total of 2000 EST sequences were produced from cDNA libraries generated from Chinese bayberry fruit (*Myrica rubra* Sieb. and Zucc. cv. “Biqi”) at four different ripening stages. After cluster and assembly analysis of the datasets by UniProt, 395 unigenes were identified, and their presumed functions were assigned to 14 putative cellular roles. Furthermore, a sequence BLAST was done for the top ten highly expressed genes in the ESTs, and genes associated with disease/defense and anthocyanin accumulation were analyzed. Gene-encoding elements associated with ethylene biosynthesis and signal transductions, in addition to other senescence-regulating proteins, as well as those associated with quality formation during fruit ripening, were also identified. Their possible roles were subsequently discussed.

## 1. Introduction

Chinese bayberry (*Myrica rubra* Sieb. and Zucc., *Myricaceae*) is a subtropical evergreen fruit tree widely grown in southern China [1,2]. For most cultivars in China, the tree blossoms in March and April, and then the fruit ripens in June and July [1]. During fruit development and ripening, chlorophylls and titratable acids decrease, while sugars increase and, for pigmented cultivars, anthocyanins accumulate rapidly, especially during the late ripening stage. Fruit at the eating-ripe stage is delicious, with total soluble solids up to 14%, titratable acid content around 1%, and anthocyanins reaching 76 mg/100 g FW for dark-purple fruit cultivars such as the “Biqi” variety [1,3,4]. In addition, bayberry is a fruit with high nutritional values, due to the high content of various bioactive compounds, as well as its significant antioxidant capacity [1,4], anti-diabetic activity [5,6], and anti-tumor activity [7]. Recently, Chinese bayberry gained more and more international attention due to its unique and attractive qualities [3,8–10]. However, the fruit has a short storage or shelf life after harvest, and fruit quality declines rapidly at ambient temperatures [4,11], thus impeding the development of the Chinese bayberry industry.

Some efforts have been made for regulating the fruit quality changes during its ripening and for controlling the postharvest fruit decay [12–14]. However, knowledge about the molecular mechanism underlying the fruit quality formation and its regulations is quite limited, which prevents the further development of the bayberry industry.

As a valuable tool for various genome-scale experiments, expressed sequence tags (EST) have been extensively applied to identify functional genes, to reveal gene expression patterns for various tissues of different developmental stages, to explore the EST-based markers, and to analyze genomes from different species [15–17]. Recently, EST-based analysis of genes associated with fruit ripening and senescence or fruit quality changes has been carried out on various woody fruit crops, such as citrus [18,19], apple [20–22], grape [23,24], kiwifruit [25], peach [26], *etc.* The results suggested that EST datasets are a valuable source for the studies associated with fruit quality regulation and for the development of functional molecular markers.

In the present study, cDNA libraries of fruit at four different ripening stages were constructed, and a total number of 2000 ESTs were obtained. Furthermore, genes related to fruit quality and ripening were identified and their transcript abundance during fruit ripening was analyzed.

## 2. Results and Discussion

### 2.1. Changes in Quality Attributes during Fruit Development and Ripening

**Figure 1:**
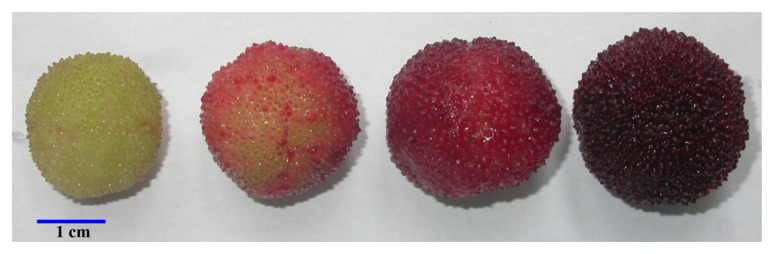
Size and color of “Biqi” fruit at mature green (MG), pink ripe (PR), red ripe (RR), and full ripe (FR) stages.

During the fruit development and ripening, significant changes took place in fruit size, weight, as well as color and flavor attributes (Figure 1 and Table 1). Fruit size and weight kept increasing from mature green (MG), pink ripe (PR), red ripe (RR), to full ripe (FR) stages during ripening, which is quite different from common fruits, such as tomato and citrus. Sugars accumulated while the organic acid content decreased during ripening, and such changes were quite apparent, especially in late ripening stages (Table 1). Fruit color development, as indicated by changes in CIRG, was well correlated with the accumulation in anthocyanins. The fully ripe fruit had an average diameter of 28.02 ± 1.16 mm, TSS of 12.95 ± 0.71 Brix, TA of 6.65 ± 0.80 mg/g FW, and total anthocyanins of 88.03 ± 9.24 mg/100 g FW.

### 2.2. Analysis of Bayberry Fruit cDNA Libraries and EST Library

**Figure 2:**
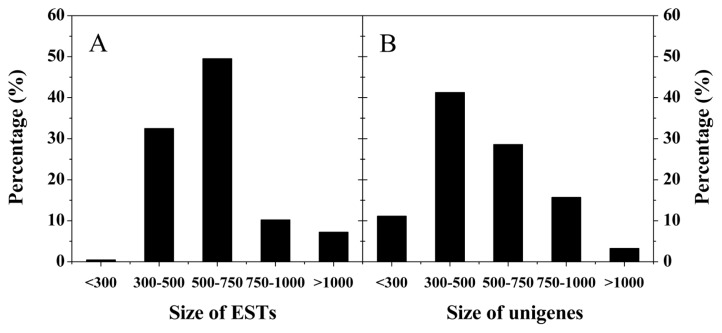
Distribution of the size of ESTs (**A**) and unigenes (**B**) of “Biqi” fruit on the basis of sequencing of 2000 recombinant clones.

The cDNA libraries for fruit at four different ripening stages were constructed with the titer of all the original cDNA libraries higher than 10^5^ cfu/mL, while the titer for final EST libraries constructed thereafter were more than 10^9^ cfu/mL (Table 2). The recombinant rates of all the libraries were over 94%, and 500 recombinant clones from each library were chosen for sequencing for EST library construction. The length of EST ranged from 300 bp to 2000 bp (Table 2), and the majority of the EST had a sequence length between 300 and 750 bp (Figure 2A). Cluster analysis showed that 395 unigenes were obtained from the 2000 ESTs, and the unigenes’ length distribution was shown in Figure 2B. The total number of tentative consensus sequences (TCs) was 94, and the total number of singletons was 301 (Table 2). The diversity of expressed mRNA was lowest for MG fruit and highest for PR fruit (Table 2), indicating that remarkable physiological changes occurred during the initiation of fruit ripening (Figure 1).

### 2.3. Functional Annotation of Bayberry EST

**Figure 3:**
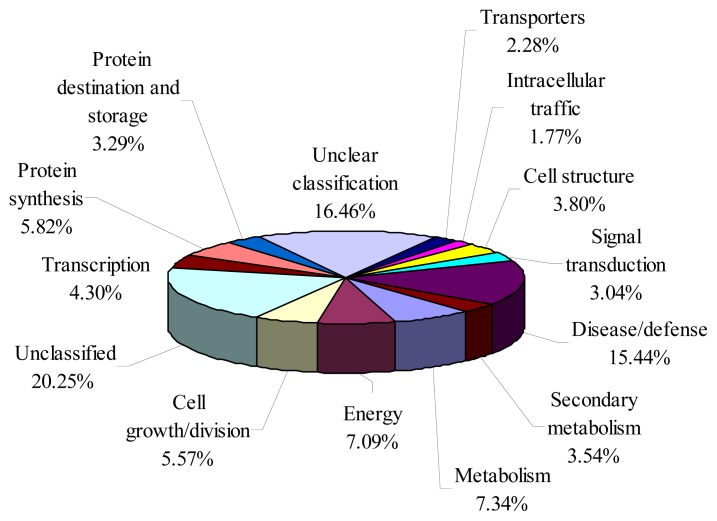
Functional classification of 395 unigenes from “Biqi” fruit EST library.

BLASTing Uniprot database with the sequences of 395 unigenes from bayberry EST resulted in functional classification of 315 unigenes, while 80 unigenes were unclassified (Figure 3). About 15.44% unigenes were related to disease/defense, and they ranked as the first group with specific functional annotation available. Genes involved in metabolism (7.34% of the total unigenes) and energy (7.09%), ranked second and third, respectively. The rest of genes were annotated and classed into various cellular events, including protein synthesis (5.82%), cell growth and division (5.57%), transcription (4.30%), cell structure (3.80%), secondary metabolism (3.54%), protein destination and storage (3.29%), signal transduction (3.04%), transporters (2.28%), and intracellular traffic (1.77%). Besides this classification of unigenes, 16.46% genes were found without clear functional denotation. Generally, as the fleshy fruit ripens, degradation of cell wall materials exacerbated, which facilitated infection of fungus pathogens. The expression of a high number of disease/defense genes might be adaptive responses of Chinese bayberry ripe fruit to environments.

### 2.4. Analysis of Highly Expressed Genes from “Biqi” Fruit ESTs

Ten highly expressed genes in the ESTs accounted for 1140 sequence reads, which was 57% of the total. The highest frequency gene (MRU00001) sequenced 408 times in four libraries, which alone accounted for 20.4% of the total EST numbers, and it showed highest amino acid sequence identity (77%) with metallothionein-like protein type 3 (MT3) from *Musa acuminata*. MTs are proteins involved in metal detoxification and homeostasis in the plant, and another two highly expressed genes (MRU00007 and MRU00032) showed 82% and 77% amino acid identity with MT2 from *Ricinus communis* and *Fagus sylvatica*, respectively. Another three highly expressed genes with more than 100 sequence reads might encode proteins, such as phytocystatin cysteine proteinase inhibitor 1 (MRU00012), phase-change related protein precursor (MRU00135), and thaumatin-like protein (MRU00040), respectively. In addition, genes encoding GAST-like protein and heat-shock proteins were all detected as highly expressed genes in “Biqi” fruit ESTs, and, therefore, data in Table 3 was consistent with those in Figure 3 for the high percentage of disease/defense genes in ESTs. The presence of gene-encoding flavonoid 3′-hydroxylase (F3′H) (MRU00008) as highly expressed genes was also consistent with the accumulation of pigment during fruit development and ripening (Table 3). Moreover, this is also consistent with what was reported on the highest transcript abundance of *MrF3′H* among all anthocyanin biosynthesis genes [27].

### 2.5. Identification of Genes Regulating Bayberry Fruit Ripening and Senescence

Based on the BLAST result, ESTs encoding 1-aminocyclopropane-1-carboxylic acid oxidase (ACO) (MRU00059), ethylene response factor (ERF) (MRU00110), ethylene-insensitive 3-like protein (EIL) (MRU00129), and 9-*cis*-epoxycarotenoid dioxygenase (NCED) (MRU00088) were identified in the ripening bayberry library (Table 4), indicating regulation of the plant hormone ethylene and ABA signal transduction pathway during fruit ripening and senescence. Chinese bayberry fruit was classified as a climacteric fruit due to its typical climacteric respiratory and ethylene behavior during postharvest storage [11]. The three ESTs identified that are associated with ethylene biosynthesis and transduction may provide important tools for the discovery of the molecular regulatory mechanisms of bayberry fruit by ethylene. Other senescence-associated proteins (MRU00062 and MRU00387), ascorbate peroxidase (MRU00242), and cytochrome C reductase protein (MRU00050) were also identified as a result of the BLAST analysis.

### 2.6. Identification of Genes Associated with Quality Formation in Ripening Bayberry Fruit

Bayberry fruit undergoes significant quality changes during fruit development and ripening [11]. Fruit pigments and volatiles accumulate as fruit become soft, juicy, and full of flavor. Over 100 ESTs were discovered to be related with such fruit quality formation and regulation. Genes encoding flavonoid 3′-hydroxylase (F3′H, MRU00008) and transcript factor MYB (MRU00113) were highly expressed ESTs during ripening stages (Table 5), which is important for the pigment accumulation during fruit development (Figure 1), and UDP-glucose:flavonoid 3-*O*-glucosyltransferase (UFGT) (MRU00098) was identified in the FR fruit library. F3′H and UFTG were proven to be two key enzymes in anthocyanin biosynthesis in Chinese bayberry, which was regulated by MrMYB1 [27].

Metabolism of sugar and organic acid during fruit development is important for the formation of flavors of bayberry characteristics [11]. Genes encoding vacuolar ATP synthase subunit G1 (MRU00009), 6-phosphogluconate dehydrogenase (MRU00061), α-glucosidase-like protein (MRU00369), and β 1–3 glucanase (MRU00046) (Table 5) were identified. Vacuolar ATP complex was important in the transport of organic acid between mitochondrion and cytoplasm, so expression of its subunit G1 was closely related to degradation of organic acid during fruit ripening.

In addition, enzymes involved in cell wall modification play an important role in regulating fruit texture changes as fruit softens during ripening. ESTs encoding polygalacturonase inhibitor-like protein (PGIP) (MRU00130), polygalacturonase (PG) (MRU00124), and peroxidase (POD) (MRU00022) were identified in the FR library (Table 5).

**Figure 4:**
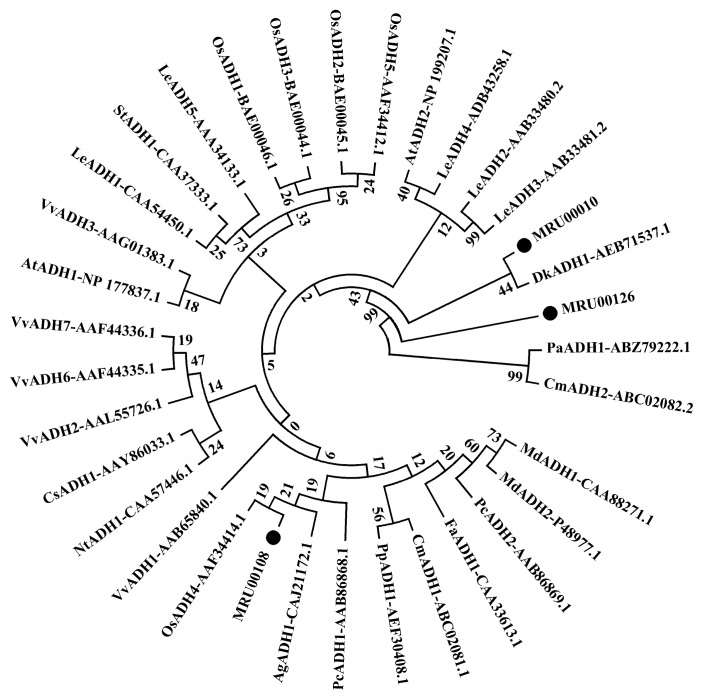
Alignment of alcohol dehydrogenase (ADH) gene family members of Chinese bayberry with other plant ADHs based on amino acid sequences.

For the aroma formation during bayberry development, 19 ESTs were identified as possibly encoding three members of alcohol dehydrogenases (ADH) (MRU00010, MRU00108, MRU00126) (Table 5). The biosynthesis of ADH during RR and FR stages was associated with the increased contents of ethanol and acetaldehyde as parts of aroma composition during fruit ripening (Table 1). Amino acid sequence alignment of three bayberry ADH members with those from other plants is shown in Figure 4, and they demonstrated high amino acid identity with ADHs from persimmon (*Diospyros kaki*), alder (*Alnus glutinosa*), and grape (*Vitis vinifera*). Additionally, ESTs encoding short chain alcohol dehydrogenase were also obtained (Table 5).

## 3. Experimental Section

### 3.1. Plant Materials

Chinese bayberry (*Myrica rubra* Sieb and Zucc. *cv.* Biqi) fruit were harvested from the Germplasm Collection of China Bayberry at Yuyao city, Zhejiang Province, China, at four different ripening stages according to fruit color, *i.e.*, mature green (MG), pink ripe (PR), red ripe (RR), and full ripe (FR) (Figure 1). The flesh tissues were taken and frozen in liquid nitrogen, and then stored at −70 °C.

### 3.2. Fruit Surface Color Measurement

Ten fruits for each ripening stage were subjected to color measurement with MiniScan XE Plus (HunterLab, Reston, VA, USA) and the color index of red grapes (CIRG) was calculated to indicate the fruit maturity as described by Zhang *et al.* [11], where *CIRG* = (180 − *H*)/(L* + *C*).

### 3.3. Total Soluble Solid (TSS) and Titratable Acidity (TA)

Total soluble solids (TSS) and total titratable acids were measured according to Zhang *et al.* [11]. TSS contents (∘Brix) of 10 fruits, two measurements per fruit, were determined with a handheld refractometer (ATAGO PR-101α, Tokyo, Japan). For TA analysis, one gram of fruit mesocarp tissue derived from a segment of flesh was ground with 5 mL of distilled water. After filtration and centrifugation for 10 min at 10,000 × *g*, the supernatant was brought to 10 mL with distilled water. The water was heated for 5 min at 100 °C to eliminate CO_2_, and subsequently titrated with freshly prepared 10 mmol/L NaOH to pH 8.2. TA was quantified as citric acid equivalents and results were expressed as mg/fresh weight (FW). The samples for TA analysis were taken in triplicate.

### 3.4. Determination of Total Anthocyanin Contents

Anthocyanin quantification was performed as described by Zhang *et al.* [4]. The fruit extract was diluted with buffers at 1:5, respectively. Absorbance at 510 nm and 700 nm using a spectrophotometer (DU-8000 Beckman Coulter, Fullerton, CA, USA) were recorded for reactions at both pHs. Results were expressed as mg C3G equivalents/100 g FW using a molar extinction coefficient of 29,600. The samples for anthocyanin determination were taken in triplicate.

### 3.5. Determination of Alcohol Contents

The method for measurements of acetaldehyde and ethanol production was slightly modified from that of Ke and Kader [28]. Frozen fruit powder was homogenized in 5 mL saturated NaCl solution. 5 mL of the mixture were put in a 10 mL air-tight test tube with crimp-top caps. Before measurement, the test tube was incubated at 60 °C for 1 h in a water bath. Then, 1 mL sample of the head space gas was withdrawn from each test tube and injected into the gas chromatograph (Lunan Chemical Engineering Instrument Co. Ltd., model GC-6800, Shandong, China) equipped with a flame ionization detector (FID) and a PGE-20K packed column (Lunan Chemical Engineering Instrument Co. Ltd). The injector, detector and oven temperatures were 150, 80 and 150 °C, respectively. Sec-butyl alcohol was added to each vial as an internal control. Acetaldehyde and ethanol was identified by comparison of retention times, and the results were calculated using standard curves.

### 3.6. Construction of Bayberry Fruit cDNA Library

Total RNA was extracted according to our previously published protocol [29]. Poly(A) RNA was isolated using PolyATtract^®^ mRNA Isolation Systems (Promega, Madison, WI, USA) according to the procedure recommended by the manufacturer, and the concentration of poly(A) RNA isolated was quantified fluorometrically as described by Wang *et al.* [30]. Synthesis of double-stranded cDNA, construction and titration of cDNA libraries were carried out using a Creator™ SMART™ cDNA Library Construction Kit (Clontech Laboratories, Inc., Mountain View, CA, USA) according to the instructions of the manufacturer, except that the cDNA size fractionation was completed with a gel-recovery strategy using Wizard^®^ PCR Preps DNA Purification System (Promega, Madison, WI, USA). The cDNA libraries were constructed using the pDNR-LIB vector. The recombinant pDNR-LIB plasmids harboring cDNAs were transformed into ElectroTen-Blue^®^ Electroporation Competent Cells (Stratagene, La Jolla, CA, USA).

The size of insert in individual cDNA clones was analyzed by conventional PCR with pDNR-F3 (5′-ACGACTCACTATAGGGCGCT-3′) and pDNR-R2 (5′-GCGCCAAACGAATGGTCTAG-3′) as primers. Clones with insert shorter than 300 bp were excluded from further sequencing. Sequencing was completed by Invitrogen Biotechnology Co., Ltd. (Shanghai, China) with pDNR-F3 as sequencing primer.

### 3.7. Bioinformatics Analysis

After removal of vector sequences, the final sequences were recorded as ESTs. Alignment of ESTs was completed with Clustal X 1.81 (Institut de Genetiave et de Biologie Moleculaire et cellvlaire, CNRS/INSERM/VLP, Illkirch Cedex, France). Sequences with a 40-bp-overlap of no less than 97.5% homology were regarded as tentative consensus sequences (TCs), while the others as singletons. Homologous information for individual unigene was obtained by BLASTing UniProt [31,32]. The function of each individual unigene was classified according to Bevan *et al.* [33].

### 3.8. Statistic Analysis

Experiments were performed in triplicate and data were expressed as the mean ± standard deviation.

## 4. Conclusions

In conclusion, based on analysis of 2000 ESTs from cDNA libraries of bayberry fruit at four different ripening stages, a total of 395 unigenes were obtained. Genes encoding elements associated with ethylene biosynthesis and signal transductions, and other senescence-regulating proteins, as well as those related to fruit quality attributes were identified. It was then observed that expression of these genes generally increased as the fruit ripened, which was suggested to be involved in fruit quality formation and regulation during fruit ripening.

## Figures and Tables

**Table 1:** Quality attributes of “Biqi” fruit at mature green (MG), pink ripe (PR), red ripe (RR), and full ripe (FR) stages.

Quality attributes	MG	PR	RR	FR
Length * (mm)	18.86 ± 0.68	20.00 ± 0.90	25.44 ± 1.75	26.47 ± 1.19
Diameter * (mm)	19.92 ± 0.42	21.65 ± 1.12	27.19 ± 1.06	28.02 ± 1.16
Weight * (g)	3.91 ± 0.42	4.90 ± 0.63	9.45 ± 1.30	11.29 ± 1.47
Color * (CIRG)	1.18 ± 0.14	2.59 ± 0.35	3.61 ± 0.29	6.01 ± 1.03
TSS * (∘Brix)	8.76 ± 0.23	9.97 ± 0.44	11.80 ± 0.75	12.95 ± 0.71
TA ** (mg/g FW)	47.18 ± 0.55	30.87 ± 0.43	6.87 ± 0.95	6.65 ± 0.80
Total anthocyanins ** (mg/100 g FW)	0.25 ± 0.12	2.67 ± 0.37	63.06 ± 16.01	88.03 ± 9.24
Ethanol ** (μmol/100 g FW)	0.76 ± 0.46	3.21 ± 0.52	6.96 ± 2.53	62.92 ± 4.99
Acetaldehyde ** (μmol/100 g FW)	1.33 ± 0.05	1.46 ± 0.07	1.48 ± 0.10	3.38 ± 0.24

* Data are expressed as means ± SD, *n* = 10;

** For titratable acids (TA), total anthocyanins, ethanol, acetaldehyde, *n* = 3.

**Table 2:** cDNA/EST library parameters on the basis of sequencing of 500 recombinant clones from “Biqi” fruit at mature green (MG), pink ripe (PR), red ripe (RR), and full ripe (FR) stages.

Library parameter	MG	PR	RR	FR
Titer of original cDNA library	2.01 × 10^5^	1.49 × 10^5^	3.92 × 10^5^	1.18 × 10^6^
Titer of EST library	3.13 × 10^9^	3.04 × 10^9^	3.53 × 10^9^	3.95 × 10^9^
Recombinant rate	94.74%	100%	94.74%	94.74%
Average length of EST (bp)	701	597	565	1272
Number of TCs	14	37	28	53
Number of singletons	45	202	36	79

**Table 3:** Analysis of highly expressed genes from “Biqi” fruit ESTs.

NO.	Unigene code	Highest BLAST match based on deduced amino acid sequences			Numbers of ESTs obtained				
	
		Deduced protein	Plant	Identity (%)	MG	PR	RR	FR	Total
1	MRU00001	Metallothionein-like protein type 3 (AF268393.1)	*Musa acuminata*	77	150	64	117	77	408
2	MRU00012	Phytocystatin cysteine proteinase inhibitor 1 (AY390352.1)	*Actinidia deliciosa*	74	19	11	48	65	143
3	MRU00135	Phase-change related protein (AJ271778.1)	*Quercus robur*	59	122	2	1	0	125
4	MRU00040	Thaumatin-like protein (AJ871175.2)	*Actinidia deliciosa*	79	65	25	11	13	114
5	MRU00003	GAST-like (cold-regulated LTCOR12)(EU717678.1)	*Litchi chinensis*	71	3	8	45	20	76
6	MRU00007	Metallothionein-like protein type 2 (L02306.1)	*Ricinus communis*	82	25	2	32	17	76
7	MRU00008	Flavonoid 3′-hydroxylase (F3′H) (DQ218417.1)	*Gerbera hybrida*	82	1	24	12	20	57
8	MRU00032	Metallothionein-like protein class II (AJ130886.1)	*Fagus sylvatica*	77	28	15	8	5	56
9	MRU00026	Putative tropinone reductase (AK175221.1)	*Arabidopsis thaliana*	72	1	3	25	25	54
10	MRU00028	Small molecular heat shock protein 17.5 ( EF100453.1)	*Nelumbo nucifera*	89	0	3	22	6	31

**Table 4:** Identification of genes associated with bayberry fruit ripening and senescence from “Biqi” fruit ESTs.

Processes involved	Unigene code	Highest BLAST match based on deduced amino acid sequences			Numbers of ESTs obtained				
	
		Deduced protein	Plant	Identity (%)	MG	PR	RR	FR	Total
Ethylene biosynthesis	MRU00059	1-aminocyclopropane-1-carboxylic acid oxidase (ACO) (AY211549.1)	*Vitis vinifera*	89				1	1
Ethylene signal transduction	MRU00110	Ethylene response factor (ERF) (AJ606475.1)	*Fagus sylvatica*	75				1	1
	MRU00129	Ethylene-insensitive 3-like protein (EIL) (GU981740.1)	*Citrus aurantium*	64				1	1
ABA biosynthesis	MRU00088	9-*cis*-epoxycarotenoid dioxygenase (NCED) (DQ309329.1)	*Citrus clementina*	86				1	1
Senescence and apoptosis	MRU00242	Ascorbate peroxidase (HQ661034.1)	*Dimocarpus longan*	94		1			1
	MRU00387	Senescence-related protein (FJ014471.1)	*Camellia sinensis*	77			1		1
	MRU00062	Senescence-associated protein (EU961590.1)	*Zea mays*	71				1	1
	MRU00050	Cytochrome C reductase (8.0 KD subunit) (X79274.1)	*Solanum tuberosum*	72		2	1	3	6

**Table 5:** Identification of genes associated with quality formation during fruit ripening from “Biqi” fruit ESTs.

Fruit quality attributes	Unigene code	Highest BLAST match based on deduced amino acid sequences			Numbers of ESTs obtained				
	
		Deduced protein	Plant	Identity (%)	MG	PR	RR	FR	Total
Color	MRU00008	Flavonoid 3′-hydroxylase (F3′H) (DQ218417.1)	*Gerbera hybrida*	82	1	24	12	20	57
	MRU00113	Myb transcription factor (HQ661039.1)	*Dimocarpus longan*	76			3	2	5
	MRU00098	UDP-glucose:flavonoid 3-*O*-glucosyltransferase (UFGT) (AY695815.1)	*Fragaria ananassa*	59				1	1
Flavor	MRU00009	Vacuolar ATP synthase subunit G1 (FJ906831.1)	*Jatropha curcas*	81			3	5	8
	MRU00061	6-phosphogluconate dehydrogenase (EU815934.1)	*Cucumis sativus*	87		1	1	1	3
	MRU00369	Alpha glucosidase-like protein (AB240194.1)	*Hordeum vulgare*	68			1		1
	MRU00046	β 1–3 glucanase (EU676805.1)	*Vitis riparia*	75				2	2
Texture	MRU00130	Polygalacturonase inhibitor-like protein (PGIP) (AJ515557.1)	*Cicer arietinum*	88				1	1
	MRU00124	Polygalacturonase (PG) (AY062697.1)	*Arabidopsis thaliana*	63				1	1
	MRU00022	Peroxidase (POD) (FJ473421.1)	*Litchi chinensis*	72				2	2
Aroma	MRU00010	Alcohol dehydrogenase (ADH) (JF357957.1)	*Diospyros kaki*	81			4	10	14
	MRU00108	Alcohol dehydrogenase (ADH) (AM062702.1)	*Alnus glutinosa*	94				1	1
	MRU00126	Alcohol dehydrogenase (ADH) (AF194173.1)	*Vitis vinifera*	88				4	4
	MRU00002	Short chain alcohol dehydrogenase (AY084550.1)	*Arabidopsis thaliana*	71				1	1
	MRU00315	Short chain alcohol dehydrogenase (AB192882.1)	*Solanum tuberosum*	64		1			1

## References

[1] Chen K.S., Xu C.J., Zhang B., Ferguson I.B. (2004). Red bayberry: Botany and horticulture. Hort. Rev.

[2] Zhang S.M., Gao Z.S., Xu C.J., Chen K.S., Wang G.Y., Zheng J.T., Lu T. (2009). Genetic diversity of Chinese bayberry (*Myrica rubra* Sieb. et Zucc.) accessions revealed by amplified fragment length polymorphism. HortScience.

[3] Chen K.S., Xu C.J., Zhang B., Ferguson I.B., Janick J., Paull R.E. (2008). *Myrica rubra* (Red Bayberry). The Encyclopedia of Fruit and Nuts.

[4] Zhang W.S., Li X., Zheng J.T., Wang G.Y., Sun C.D., Ferguson I.B., Chen K.S. (2008). Bioactive components and antioxidant capacity of Chinese bayberry (*Myrica rubra* Sieb. and Zucc.) fruit in relation to fruit maturity and postharvest storage. Eur. Food Res. Technol.

[5] Zhang B., Kang M., Xie Q., Xu B., Sun C., Chen K., Wu Y. (2011). Anthocyanins from Chinese bayberry extract protect β cells from oxidative stress-mediated injury via HO-1 upregulation. J. Agric. Food Chem.

[6] Sun C.D., Zhang B., Zhang J.K., Xu C.J., Wu Y.L., Li X., Chen K.S. (2012). Cyanidin-3-glucoside-rich extract from Chinese bayberry fruit protects pancreatic β cells and ameliorates hyperglycemia in streptozotocin-induced diabetic mice. J. Med. Food.

[7] Sun C.D., Zheng Y.X., Chen Q.J., Tang X.L., Jiang M., Zhang J.K., Li X., Chen K.S. (2012). Purification and anti-tumor activity of cyanidin-3-*O*-glucoside from Chinese bayberry fruit. Food Chem.

[8] Joyce D.C. (2007). Evaluation of fresh red bayberry (*Myrica rubra*) fruit acceptance. N. Z. J. Crop Hort.

[9] Karp D (2007). From China, only in a bottle, a berry with an alluring name. The New York Times.

[10] Karp D. (2008). *Myrica rubra*, a fruit of many names. Fruit Gardener.

[11] Zhang W.S., Chen K.S., Zhang B., Sun C.D., Cai C., Zhou C.H., Xu W.P., Zhang W.Q., Ferguson I.B. (2005). Postharvest responses of Chinese bayberry fruit. Postharvest Biol. Technol.

[12] Zhang W.S., Li X., Wang X.X., Wang G.Y., Zheng J.T., Abeysinghe D.C., Ferguson I.B., Chen K.S. (2007). Ethanol vapour treatment alleviates postharvest decay and maintains fruit quality in Chinese bayberry fruit. Postharvest Biol. Technol.

[13] Yang Z.F., Heng Y.H., Cao S.F. (2009). Effect of high oxygen atmosphere storage on quality, antioxidant enzymes, and DPPH-radical scavenging activity of Chinese bayberry fruit. J. Agric. Food Chem.

[14] Wang K.T., Jin P., Tang S.S., Shang H.T., Rui H.J., Di H.T., Cai Y., Zheng Y.H. (2011). Improved control of postharvest decay in Chinese bayberries by a combination treatment of ethanol vapor with hot air. Food Control.

[15] Alba R., Fei Z., Payton P., Liu Y., Moore S.L., Debbie P., Cohn J., Ascenzo M.D., Gordon J.S., Rose J.K.C. (2004). ESTs, cDNA microarrays, and gene expression profiling: Tools for dissecting plant physiology and development. Plant J.

[16] Pashley C.H., Ellis J.R., McCauley D.E., Burke J.M. (2006). EST databases as a source for molecular markers: Lessons from Helianthus. J. Hered.

[17] Rudd S. (2003). Expressed sequence tags: Alternative or complement to whole genome sequences?. Trends Plant Sci.

[18] Cercós M., Soler G., Iglesias D.J., Gadea J., Forment J., Talón M. (2006). Global analysis of gene expression during development and ripening of citrus fruit flesh. A proposed mechanism for citric acid utilization. Plant Mol. Biol.

[19] Terol J., Conesa A., Colmenero J.M., Cercos M., Tadeo F., Agustí J., Alós E., Andres F., Soler G., Brumos J. (2007). Analysis of 13,000 unique Citrus clusters associated with fruit quality, production and salinity tolerance. BMC Genomics.

[20] Janssen B.J., Thodey K., Schaffer R.J., Alba R., Balakrishnan L., Bishop R., Bowen J.H., Crowhurst R.N., Gleave A.P., Ledger S. (2008). Global gene expression analysis of apple fruit development from the floral bud to ripe fruit. BMC Plant Biol.

[21] Newcomb R.D., Crowhurst R.N., Gleave A.P., Rikkerink E.H.A., Allan A.C., Beuning L.L., Bowen J.H., Gera E., Jamieson K.R., Janssen B.J. (2006). Analyses of expressed sequence tags from apple. Plant Physiol.

[22] Park S., Sugimoto N., Larson M.D., Beaudry R., Nocker S. (2006). Identification of genes with potential roles in apple fruit development and biochemistry through large-scale statistical analysis of expressed sequence tags. Plant Physiol.

[23] Da Silva F.G., Iandolino A., Al-Kayal F., Bohlmann M.C., Cushman M.A., Lim H., Ergul A., Figueroa R., Kabuloglu E.K., Osborne C. (2005). Characterizing the grape transcriptome. Analysis of expressed sequence tags from multiple *Vitis* species and development of a compendium of gene expression during berry development. Plant Physiol.

[24] Wang X.C., Guo L., Shangguan L.F., Wang C., Yang G., Qu S.C., Fang J.G. (2012). Analysis of expressed sequence tags from grapevine flower and fruit and development of simple sequence repeat markers. Mol. Biol. Rep.

[25] Crowhurst R.N., Gleave A.P., MacRae E.A., Ampomah-Dwamena C., Atkinson R.G., Beuning L.L., Bulley S.M., Chagne D., Marsh K.B., Matich A.J. (2008). Analysis of expressed sequence tags from Actinidia: Applications of a cross species EST database for gene discovery in the areas of flavor, health, color and ripening. BMC Genomics.

[26] Ogundiwin E.A., Martí C., Forment J., Pons C., Granell A., Gradziel T.M., Peace C.P., Crisosto C.H. (2008). Development of ChillPeach genomic tools and identification of cold-responsive genes in peach fruit. Plant Mol. Biol.

[27] Niu S.S., Xu C.J., Zhang W.S., Zhang B., Li X., Lin-Wang K., Ferguson I.B., Allan A.C., Chen K.S. (2010). Coordinated regulation of anthocyanin biosynthesis in Chinese bayberry (*Myrica rubra*) fruit by a R2R3 MYB transcription factor. Planta.

[28] Ke D., Kader A. (1990). Tolerance of “Valencia” oranges to controlled atmospheres as determined by physiological responses and quality attributes. J. Am. Soc. Hortic. Sci.

[29] Xu C.J., Zhu C.Q., Gao Z.S., Chen K.S. (2009). Application of fruit crop ESTs in studies on fruit development and ripening. J. Fruit Sci.

[30] Wang W.J., Chen K.S., Xu C.J. (2006). DNA quantification using EvaGreen and a real-time PCR instrument. Anal. Biochem.

[31] The UniProt Consortium (2008). The universal protein resource (UniProt). Nucleic Acids Res.

[32] UniProt.

[33] Bevan M., Bancroft I., Bent E., Love K., Goodman H., Dean C., Bergkamp R., Dirkse W., van Staveren M., Stiekema W. (1998). Analysis of 1.9 Mb of contiguous sequence from chromosome 4 of *Arabidopsis thaliana*. Nature.

